# When to start antiretroviral therapy in resource-limited settings: a human rights analysis

**DOI:** 10.1186/1472-698X-10-6

**Published:** 2010-03-31

**Authors:** Nathan Ford, Alexandra Calmy, Samia Hurst

**Affiliations:** 1 Médecins Sans Frontières, Cape Town, South Africa; 2 Centre for Infectious Disease Epidemiology and Research, Cape Town, South Africa; 3 Médecins Sans Frontières, Campaign for Access to Essential Medicines, Geneva, Switzerland; 4 Geneva University Hospital, Geneva, Switzerland; 5 Institute for Biomedical Ethics, Geneva, Switzerland

## Abstract

**Background:**

Recent evidence from developed and developing countries shows clear clinical and public health benefit to starting antiretroviral therapy (ART) earlier. While discussions about when to start ART have often focused on the clinical risks and benefits, the main issue is one of fair limit-setting. We applied a human rights framework to assess a policy of early treatment initiation according to the following criteria: public-health purpose; likely effectiveness; specificity; human rights burdens and benefits; potential for less restrictive approaches; and fair administration.

**Discussion:**

According to our analysis, a policy of earlier ART initiation would better serve both public health and human rights objectives. We highlight a number of policy approaches that could be taken to help meet this aim, including increased international financial support, alternative models of care, and policies to secure the most affordable sources of appropriate antiretroviral drugs.

**Summary:**

Widespread implementation of earlier ART initiation is challenging in resource-limited settings. Nevertheless, rationing of essential medicines is a restriction of human rights, and the principle of least restriction serves to focus attention on alternative measures such as adapting health service models to increase capacity, decreasing costs, and seeking additional international funding. Progressive realisation using well-defined steps will be necessary to allow for a phased implementation as part of a framework of short-term targets towards nationwide policy adoption, and will require international technical and financial support.

## Background

Highly active antiretroviral therapy (ART) has transformed HIV/AIDS from a death sentence into a manageable, chronic disease. Today, an adult 20 years of age diagnosed with HIV/AIDS in the developed world can expect to live at least 23 years [1,2]. In the developing world, fewer therapeutic options are available for patients; nevertheless current treatment approaches are effective at reducing mortality, with studies demonstrating similar survival outcomes compared to western countries, at least in the short term [3].

Among the different strategies for improving long-term survival for people with HIV/AIDS in resource-limited settings, the question of when to start ART is gaining increasing attention. Studies from developed and developing country settings conclude that early initiation results in substantial gains in survival and reduced incidence of opportunistic infections, in particular tuberculosis (TB). However, a number of concerns have been put forward against starting treatment earlier, namely increased costs, potential toxicity of treating more patients longer, and increased burden on health systems.

One approach to disentangling competing policy goals in a manner supportive of patient need is through a human rights analysis [4]. Human rights analysis frameworks provide a methodology for assessing health policy from a range of different perspectives, and thus provide a broader analysis that draws on a range of disciplines, in contrast to more conventional research methods such as systematic reviews or cost-effective analyses. Such analyses have been applied to a number of different issues of importance to public health, such as the prevention of mother-to-child transmission of HIV [5] and management of drug-resistant TB [6]. This paper applies such an analysis to the question of when to start antiretroviral treatment.

## When to start antiretroviral treatment

HIV infection progresses to AIDS disease as the virus replicates in cells of the immune system (CD4 cells), destroying a patient's immune defence and allowing opportunistic infections to take hold. ART works by preventing viral replication.

The decision of when to start ART is generally made according to clinical or immunological criteria. Clinical decisions are based on the presence of one or more severe opportunistic infections, categorised by the World Health Organisation (WHO) as stage III and IV AIDS-defining illnesses. Developed and developing country guidelines all recommend starting ART if a patient presents with a stage III or IV infection, though decisions based on such clinical criteria alone are generally only used in resource-limited settings where laboratory capacity is limited. More commonly, the decision to start ART is based on immunological criteria, as defined by the level of CD4 cells.

Until recently, the level of CD4 indicating ART differed between developed and developing countries. European and US guidelines recommend ART initiation at a CD4 cell threshold of 350 cells/μL (moderate immunodeficiency). A policy of deffered initiation (200 cells/μL) was originally based on concerns related to the accumulative risks of toxicity and drug resistance [7]. Such concerns have diminished in recent years as newer medicines have become available with fewer toxicities and better potency (reducing the chance of resistance development). The availability of these newer medicines, together with studies that have increased the understanding of the risks of developing life-threatening illnesses over time if ART is initiated at a low CD4 count, have shifted the risk-benefit equation [7]. Recent evidence from European cohorts showed that starting ART earlier (at least 350 cells/μL) results in significant survival gains [8].

Guidelines for developing countries have recently been revised in line with developed world recommendations. Treatment guidelines issued by the International AIDS Society in August 2008 state that "the core principle underlying these guidelines, namely pathogenesis-directed therapy with regimens designed to achieve full virologic suppression with minimal toxicity and maximal simplicity, is applicable to the developing world" [9], and the latest WHO antiretroviral treatment guidelines for resource-limited settings released at the end of 2009 recommend a move towards earlier initiation at CD4 count <350 cells/μL [10].

However, these recommendations have for the most part yet to be translated into country-level policy, and most national guidelines in developing countries continue to recommend "deferred" ART initiation at CD4 <200 cells/μL (severe immunodeficiency). The main concern for developing countries is that providing treatment earlier, for longer, would increase overall drug expenditure costs [11]. In addition, other significant health systems costs are associated with ART provision to large numbers of people when there are already too few doctors and hospitals are saturated [12]. Finally, given that most people present at ART services with an even lower CD4 count of around 100 cells/μL [13], the issue of early initiation has been argued to be a moot point. These issues have led some to voice concern that earlier initiation "may end up doing more harm than good" by weakening already strained ART programmes [14].

## Discussion

A number of frameworks have been developed to assess the impact of public health policy on human rights. We chose the Mann-Gostin Framework [4] because it is well suited to the analysis of policies that involve a restriction of rights, which can be considered to be the case when ART is deferred. This framework interrogates policy according to the following questions: what is the public-health purpose of the policy? What is the likely effectiveness of the policy in relation to its purpose? Is the policy well targeted? What are the human rights burdens and benefits? Is there a less restrictive policy to achieve the same objective? Are there fair administrative procedures in place? These considerations are discussed below and summarised in Additional File 1, Table S1.

### What is the public-health purpose?

When analysing the public health purpose of a given policy, the objectives of the policy should be assessed independently of the methods chosen to reach them. The three main reasons for establishing a restrictive threshold for initiating ART are: (i) to treat people who need ART; (ii) to minimise harms caused by prolonged exposure to toxicity; and (iii) to ration care.

The primary goal of ART is to decrease HIV-related morbidity and mortality. The benefits of starting ART in terms of reduced mortality and morbidity are clear, and these are most immediately evident when a patient's immune system is severely compromised, although a patient's immunological nadir - how low their CD4 count is allowed to drop - is predictive of how successfully future ART will benefit them [15].

A related health concern, which was the principal reason for delaying ART in developed countries, is the risk of accumulated drug toxicities. The main drugs used in Africa for first-line ART - stavudine, zidovudine, lamivudine, efavirenz, and nevirapine [16] - all have associated toxicities, including nausea, diarrhoea, and headache; more severe adverse effects such as acute hepatitis, anaemia, neuropathy, lipodystrophy, hypersensitivity, and pancreatitis; and life-threatening toxicities such as fulminant hepatitis and lactic acidosis [17].

However, the overriding purpose of maintaining a more restrictive threshold for initiation is unstated in policy: it acts as a form of rationing treatment. Rationing can be defined as "any implicit or explicit mechanisms that allow people to go without beneficial services" [18].

### What is the likely effectiveness of the policy?

With lower CD4 count comes higher risk of mortality and morbidity. In untreated patients with a high viral load, the risks of developing AIDS within 6 months are approximately 40%, 10%, and 3% for CD4 cell count groups <200, 200-349, and >350 cells/μL, respectively [19]. Similarly, the long-term prognosis for patients on ART is determined by immune status at initiation: patients starting ART at a lower CD4 count have a lower chance of long-term survival [20].

A recent randomised trial conducted in Haiti comparing patients who started ART early (<350 cells/μL) and those who were deferred (<200 cells/μL) found a 4-fold increase in mortality and a 2-fold increase in incident TB in the deferred group [21]. This reduction in TB incidence also suggests a public health benefit to starting earlier where high coverage can be achieved. These data reinforce evidence from trials in Africa suggesting a greater survival benefit of starting therapy earlier [22].

While the focus to date has been on opportunistic infections that are most frequent at CD4 <200 cells/μL, recent studies have raised concern about the risk of death from liver, renal, and heart diseases, as well as from "non-AIDS" cancers; incidence of these diseases is increased at lower CD4 counts, with significant differences seen between those with CD4 <350 cells/μL and those with CD4 >350 cells/μL [23]. Therefore, if reducing mortality and morbidity is the main objective, then on the basis of the latest clinical evidence the 350 cells/μL threshold should be adopted everywhere. HIV-positive pregnant women should be provided with ART regardless of CD4 count.

Furthermore, models have indicated a public health benefit in terms of reduced transmission. A modelling study from South Africa found a 54% reduction of HIV transmission when therapy is initiated at CD4 <350 cells/μL as compared to <200 cells/μL [24]. The potential for broader access to reduce HIV transmission was suggested by subsequent studies [25].

Concerns about long-term toxicity and drug resistance have been lessened in the developed world as more potent and less toxic medicines became available [26-28]. These concerns are still justified in developing countries, where older drugs with less favourable side-effect profiles still form the backbone of therapy, but would be largely overcome with the wider availability of less-toxic drugs. Withholding medicines is certainly an effective way of preventing the development of toxicity and resistance in the short term, but in the long term everyone will eventually be eligible. Delaying treatment until the CD4 count has fallen below 300-350 cells/μL carries greater risks than does starting treatment earlier, provided that less toxic drugs are available [7].

Rationing care can be viewed as having two legitimate aims: getting more benefit from available resources by giving priority to more cost-effective interventions over less cost-effective ones targeting the same condition, and allocating available health resources as fairly as possible. In the first sense, using a low CD4 count as a means to ration ART could be counterproductive. Symptomatic patients place the greatest burden on health systems, as they require multiple doctor consultations and hospitalisation. In the same way that provision of ART led to massive cost savings in terms of avoided hospitalisations and opportunistic infections [29], the cost savings made by delaying ART initiation are at least partly offset by the cost of treating opportunistic infections among those who present sick (with low CD4). A cost-effectiveness simulation from South Africa found that earlier initiation would be cost-effective over a 5-year period [30]. This means that a restrictive CD4 threshold for treatment initiation will also be counterproductive in attempting to allocate resources fairly; "doing less with more" in this area also implies depriving others of needed interventions towards which these resources could otherwise have been channelled.

Moreover, even at the lower cut-off of CD4 <200 cells/μL, still less than half of those eligible for treatment in the developing world are receiving ART. It must therefore be acknowledged that CD4 count is not the only criterion, or even the main one, by which care is currently being rationed. Lack of capacity within health systems and poor proximity to health-care entry points play much greater roles [31].

### Is the policy well targeted?

Deferred treatment initiation prioritises those patients in greatest clinical need of ART, but still excludes substantial numbers of patients at risk. Given that the latest evidence supports initiating treatment at CD4 <350, a cut-off below that level is a decision to treat some, but not all, of the patients who would substantially benefit. Therefore, a policy of deferred initiation can at best be considered to be moderately well targeted.

In terms of limiting toxicity, the policy is poorly targeted. Although it limits the overall person-time exposure to ART, not all toxicities are cumulative. As everyone who is HIV-positive will eventually need to be put on treatment and so will be exposed, the policy only delays exposure to toxicity, but does not address the underlying causes: the fact that toxic drugs are being provided when less toxic alternatives exist.

A major challenge for ART programmes in resource-poor settings is pre-ART defaulting: patients diagnosed as HIV-positive but not yet eligible for treatment fall out of care because they have little reason to visit the clinic. One recent study from South Africa reported that almost three-quarters of patients defaulted pre-ART [32]. While an argument in favour of a lower CD4 threshold could be that these individuals would be eligible for ART once their CD4 count has descended to below 200 cells/μL, the reality is that many of these patients are not seen again until they are very sick. Raising the threshold could support this goal by providing more opportunities to enrol people into treatment and retain them in care before they become sick.

### What are the human rights benefits and burdens?

The United Nations Commission on Human Rights explicitly recognised "that access to medication in the context of pandemics such as HIV/AIDS is one fundamental element for achieving progressively the full realisation of the right of everyone to the enjoyment of the highest attainable standard of physical and mental health" [33]. This right is subject to both progressive and immediate realisation. Article 2 (1) of the International Covenant on Economic, Social and Cultural Rights (ICESCR) stipulates the right to the highest attainable standard of health, including access to medicines, is subject to progressive realisation and resource availability [34]. At the same time, General Comment 14 of the UN Committee on Economic, Social and Cultural Rights (CESCR) declares that states have an immediate obligation to make essential medicines available and accessible throughout their jurisdiction [35]. Antiretroviral drugs are defined as essential medicines [36].

Rationing of ART brings into consideration a number of human rights principles that are articulated by international and regional human rights instruments. The most relevant concerns covered by these instruments include the right to life [34,36-38], access to health care [34,36-38], access to medicines [33,34,37], non-discrimination [34], protection of the most vulnerable [34], and restriction of rights [37,39,40].

A number of benefits and burdens are associated with the policy of using a restrictive CD4 count to determine ART eligibility. The main issues are outlined below.

#### Benefits

As a general point, from a human rights perspective, rationing of ART could bring a benefit if it led to broader access, and thus to greater overall implementation of the rights to life, access to health care, and access to medicine. This, however, would only be the case if limiting indications enabled health systems to provide access more extensively to persons who would otherwise be deprived of it. For example, this could apply if rationing was required to make ART available to everyone who met a lower CD4 threshold, even in rural areas, rather than to all who presented with the higher threshold, but exclusively at urban centres. Moreover, such a benefit could only ever be said to exist in circumstances where full access for all was not feasible. More specifically, one of the most important aspects of employing medical criteria, as opposed to other forms of rationing such as occupation or social worth [41], is that it meets the criteria of non-discrimination [42]: everyone meeting the criteria is given a chance to access treatment via the consistent application of the same criteria in an objective and transparent manner.

There is, however, a concern regarding the fact that eligibility based on laboratory investigations requires access to those investigations. While clinical criteria would determine that a certain proportion of patients should start treatment, those who would be eligible on immunological but not clinical grounds would be denied care. They are in any case likely to be among the most vulnerable, and in some cases this difference in treatment will even amount to a form of inequity in settings where CD4 is poorly available [41]. However, this applies equally to any CD4-based initiation strategy, whether early or deferred. Certainly, CD4 counts provide a more objective threshold than clinical criteria.

#### Burdens

The main human rights burden of limiting ART is that it denies treatment to people who, in other (wealthier) parts of the world, would be considered eligible. If access to ART is subject to immediate realisation, and the latest evidence suggests that ART should be provided earlier, then from a human rights perspective every effort should be made to ensure this happens and happens for all. Higher CD4 count at treatment initiation gives a greater chance of escaping symptomatic disease. Crudely put, the current policy is one that lets people progress from having a 10% chance of developing AIDS illness within 6 months to a 40% chance. This is in clear conflict with the human right to the highest attainable standard of health [34].

Finally, the issue of stigma is important. The common symptoms of AIDS-defining illnesses are well-known within communities, and allowing people with HIV to develop symptoms may increase their risks of being stigmatised [43]. This represents a violation of the right to non-discrimination [34]. Stigma can also act as a barrier to uptake of HIV services [44] and as such goes against the right to access to health care.

### Is there a less restrictive policy to achieve objective?

Human rights doctrine recognises the need to limit certain human rights, usually in times of public health emergency when certain individual rights are temporarily restricted over concern for the common good. The most commonly cited example of such a trade off is the isolation of individuals to prevent the spread of infectious diseases. A core principle of such restrictions is that they are legitimate, non-arbitrary, and necessary. The Siracusa Principles on the Limitation and Derogation of Provisions in the International Covenant on Civil and Political Rights state that: "Public health may be invoked as a ground for limiting certain rights in order to allow a state to take measures dealing with a serious threat to the health of the population or individual members of the population. These measures must be specifically aimed at preventing disease or injury or providing care for the sick and injured" [45].

Human rights considerations require that where different policy options may be pursued to reach the same objective, the less-restrictive policy should be applied. In terms of meeting the stated public health objectives of reducing mortality and morbidity, increasing the threshold to 350 cells/μL would be less restrictive. In regards to limiting exposure to toxic drugs, a number of medicines are available today with a better toxicity profiles than those most widely used in the developing world. One of the most severe side effects, lactic acidosis, can be largely avoided by replacing one drug (stavudine) with less toxic alternatives (tenofovir or abacavir). However, these drugs are currently more expensive and widespread adoption will likely require a reduction in price [46]; of note, mechanisms which have been successful in influencing drug prices and encouraging generic competition also include the use of public health safeguards [47-49]]. Thailand and Brazil, for example, have both issued compulsory licenses to enable them to purchase generic versions of tenofovir, which is more affordable than the patented version [48]. The Indian patent office recently rejected the patent on tenofovir, allowing generic production [49]. The global price for tenofovir has fallen commensurate with an increase in generic production [28].

Any policy to ration essential care is by definition restrictive, and such restrictions are a feature of health care across the world. However, policies that restrict essential health services on the basis of limited resources have been challenged elsewhere. In the UK for example, a high court decision regarding the rationing of leukaemia chemotherapy ruled that the health authorities' power to refuse treatment on grounds of resource shortage were severely limited, and that the authorities had to prove that the money saved by rationing was being better placed elsewhere [50]. Although made in a developed country, this ruling makes the general point that if rationing is being employed, it is incumbent on the state to demonstrate that resources saved in one area are meeting important priorities elsewhere.

Recent cuts in the HIV/AIDS budgets in some developing countries suggest that this is not the case [51]. In the case of HIV/AIDS treatment, a substantial portion of funding comes from the international community, notably developed countries who do not themselves apply such limiting criteria. Although most human rights documents address the duties of states towards their own citizens only, this is a case where, arguably, this limitation does not fully apply. As pointed out by UN Committee on Economic, Social and Cultural Rights "...given that some diseases are easily transmissible beyond the frontiers of a State, the international community has a collective responsibility to address this problem. The economically developed States parties have a special responsibility and interest to assist the poorer developing States in this regard" [29]. HIV/AIDS clearly falls into this category. It is therefore also incumbent on donor countries to justify why they do not support a policy of earlier initiation.

Part of the justification for rationing is based on the fact that current ART sites are overburdened. Much could be done to increase capacity by increasing efficiency, which would be a less restrictive alternative to rationing. Various national and international guidelines recommend ways of doing this. One such measure is the task-shifting of clinical responsibilities from doctors to nurses and the deployment of community health workers to overcome severe human resource shortages [52]. There are also emerging decentralised models of how to manage "stable" patients out-of-facility [53]. Such approaches provide alternative ways of improving cost savings and increasing access despite bottlenecks due to doctor shortages, and are consistent with health-systems approaches supporting the right to the highest attainable standard of health [54].

Finally, international financing already supports a substantial part of the AIDS response [55]. This is not mere charity on the part of wealthier countries, but is consistent with their obligation under international human rights law to provide resources to support the realisation of "core and other obligations," which includes access to ART [56].

### Are fair administrative procedures in place?

A human rights-based approach to HIV care means ensuring transparency and accountability for how policies and programmes are carried out. This relates to a state's duty to promote human rights by providing rights holders with sufficient information to realise their rights [57]. The requirement for fair administrative procedures means that the policy - in this instance, to ration ART by CD4 count, or relying on clinical criteria where CD4 measures are not possible - is based on adequate assessment of all relevant evidence and that safeguards are in place to provide opportunities for appeal and review, and that all parties understand the reasons behind the decisions taken [58]. Most national guidelines in developing countries do not adequately reflect the latest evidence regarding the clinical and public health benefits of initiating therapy at the higher threshold of CD4 <350 cells/μL. Moreover, it is unlikely that the decision to ration treatment in this way is properly understood by health providers and recipients (patients). In particular, the high rate of defaulting among HIV-positive patients who are not yet eligible for ART suggests that many do not understand the need to remain in care until eligibility requirements are met.

### Overall, is the policy the optimal approach to the problem?

From this analysis, the policy is suboptimal in terms of addressing the public health and human rights issues in terms of reducing mortality and morbidity, limiting toxicity, supporting public health goals, and rationing.

### Alternative frameworks for assessing rationing decisions

It has been argued that "No single analytical framework - whether grounded in economics, social sciences, ethics or human rights - can determine to everyone's satisfaction who should benefit from services first, second or last."[57]

Human rights principles of restriction of rights have most often been looked at from a perspective of restriction of movement [5] but could also be employed to analyse rationing decisions. Ethics and human rights are interconnected; both are based on the core principles of the respectful and dignified treatment of persons [58]. Since the inclusion of a right to health within the Human Rights framework, both also recognise a right to health care. Currently, the most influential ethical defence of such a right is based on the fact that health is a prerequisite if we are to have access to the life choices which should be open to everyone [59-61]. Although a full discussion is beyond the scope of this paper, this is arguably particularly relevant to access to ART, as without it patients are vulnerable to stigma, disability, and death All three effectively close options that these patients should have, and which treatment can reopen. Inasmuch as stigma compounds other sources of vulnerability, this may even constitute a reason to give a degree of priority to ART in health care resource allocation [62].

Discussions of ethical aspects of rationing have focused on individual-level access and decisions, as well as institutional or national priorities in resource allocation [63]. These discussions have however tended to focus less on public-health interventions than on clinical or other health policy decisions. Two recent examples from the US - oseltamivir and influenza vaccine - have looked at rationing from a public health perspective. In the case of oseltamivir (an antiviral drug for the treatment of influenza) it was argued that while, in general, doctors should defer to patients' requests for a treatment that provided a modicum of benefit, where the drug is limited in quantity, physicians should be guided by principles of public-health ethics (maximising the health of the population while minimising infringements on individual liberty) [64]. Public discussions about rationing were limited for fear of evoking panic, allowing "invisible" rationing decisions to be made [65]. Similar considerations were brought to bear in the case of influenza vaccine, where rationing was accepted by health professionals and doctors as a necessary way to manage limited resources [66]. However, broader decisions about resource allocation were based on prioritising key personnel that by definition excluded those who were unemployed or in "nonessential" jobs. While this can ensure greater protection for a broader public, it can also exclude the most disenfranchised populations, thereby promoting social injustice [67].

Both of these issues - lack of discussion and the choice of prioritisation criteria - are ethically problematic. The main point emphasised in both these examples is the need for public discourse to ensure transparency in decision-making. This has become an increasing focus of ethical discussions as attempts to identify generally justifiable criteria for allocation failed, and has led to the development of philosophical frameworks for procedural justice in resource allocation [68]. This need is broadly recognised: when the recent trial data showed the benefits of earlier initiation, patient groups in South Africa immediately demanded a change in guidelines [69]. Nevertheless, the involvement of people with HIV/AIDS in such decisions is at best limited to public advocacy; they are rarely invited to the policy table. Thus, a need exists to explicitly frame and debate the main issues at stake by engaging key stakeholders from all sides - government, providers, civil society, and patients. Such debate should not be limited to narrow cost concerns but should consider broader principles that apply to rationing policies such as justice, reciprocity, consistency, explicitness, and revisability [70]. Public debate is also needed to gain political support for moving towards a policy that would effectively result in a drop in ART coverage as the number of people in need of treatment will increase.

## Summary

The latest evidence on risks to health for people with HIV at CD4 counts above 200 cells/μL, together with the availably of newer, less toxic drugs, support the raising of the threshold of ART initiation from public health, ethical, and human rights perspectives. Guidelines for developed countries have adopted a higher cut-off for ART eligibility, and international guidelines for developing countries have recently been amended to support earlier initiation. However, most national-level guidelines continue to recommend deferring treatment to 200 cells/μL, a policy that, in human rights terms, falls into the category of "impermissible under-inclusiveness" [4].

This paper aims to show that a human rights analysis can contribute to more considered deliberation regarding the way forward, from which concrete policy options can emerge (Additional File 2, Table S2). The framework used here serves to clarify a number of important issues. The first step was to clarify the public health purpose. Clearly no medical reason exists for why deferred ART initiation should be preferred. Rather, the principal purpose of starting treatment later is to ration care. Rationing of essential medicines is a restriction of human rights, and the principle of least restriction serves to focus attention on alternative measures such as implementing policies that would increase capacity and decrease costs of care, and seeking additional funding to support ART expansion. Finally, where resources remain insufficient to provide early treatment for all, different views exist among governments, academia, civil society, and donors, and the principal of fair administration highlights the need to discuss these issues in a transparent manner involving all relevant stakeholders.

More data are needed on the cost-effectiveness of starting ART earlier, including not only the costs associated with treating opportunistic infections but also the costs of delaying ART [30]. Such evidence could be gained through the gradual adoption of an earlier threshold for initiation in pilot sites. This would also allow for important lessons to be learnt in terms of training staff, educating patients, and scaling up services. Such cost effectiveness data will be critical given that a shift towards earlier initiation will require both affected-country governments and the international community to provide additional funding to support expansion of care, even if long-term savings can be anticipated. Finally, implementation of these new recommendations is challenging. Progressive realisation using well-defined steps will be necessary to allow for a phased implementation as part of a framework of short-term targets towards nationwide policy adoption.

In conclusion, we believe that instead of continuing a policy of rationing ART based on medical criteria that have been abandoned elsewhere, governments should revise the initiation threshold in line with international recommendations, adopt a treatment policy that includes the use of less-toxic drugs, and implement cost-effective policies such as task-shifting and sourcing of more affordable medicines on the international market. Such measures require clear technical and financial support from donor governments that are currently applying a double standard by supporting ART care with restrictions to access that they themselves would not accept. Indeed, it is their duty under international law to provide such assistance.

## Competing interests

The authors declare that they have no competing interests.

## Authors' contributions

NF conceived of the study and wrote the first draft. All authors contributed to subsequent drafts. All authors have read and approved the final manuscript.

## Pre-publication history

The pre-publication history for this paper can be accessed here:

http://www.biomedcentral.com/1472-698X/10/6/prepub

## Supplementary Material

Additional file 1 **Human rights analysis of when to start antiretroviral therapy**. This table summarises the main considerations regarding different thresholds of initiation of antiretroviral therapy from a human rights and public health perspective. Click here for file

Additional file 2 **Recommendations for policy**. This table provides a summary of recommendations for policy, taking into consideration the main issues identified by the human rights analysis. Click here for file
