# Supplementary material for: When to start antiretroviral therapy in resource-limited settings: a human rights analysis
Source: BMC Int Health Hum Rights. 2010 Mar 31;10:6. doi: 10.1186/1472-698X-10-6 (PMC2864209; doi:10.1186/1472-698X-10-6)
Supplement: Additional file 1 — Human rights analysis of when to start antiretroviral therapy. This table summarises the main considerations regarding different thresholds of initiation of antiretroviral therapy from a human rights and public health perspective. [file 1472-698X-10-6-S1.DOC]

**Table S1: Human rights analysis of when to start antiretroviral therapy**

|  | **Public Health Purpose** | | |
| --- | --- | --- | --- |
|  | ***Reduce mortality and morbidity*** | ***Limit toxicity*** | ***Rationing*** |
| **Human rights analysis** |  |  |  |
| ***What is its likely effectiveness?*** | **Moderate**  - targets those at greatest risk of mortality  - promotes the development of opportunistic infections, including TB  - evidence supports an increase to CD4 <350:  i) higher likelihood of treatment success if ART initiated earlier  ii) illnesses develop even at CD4>200 | **Moderate**  **-** delays number of people exposed to ART-related toxicities but does not address primary cause (the use of toxic drugs) | **Low**  - Other mechanisms dominate |
| ***Is the policy well targeted?*** | **Yes/No**  - applies to all HIV+ people  - excludes some patients who would benefit from treatment  - misses opportunities to enrol patients who may become eligible in the future (ie does not address pre-ART defaulting) | **Yes/No**  - limits person-time exposure to ART  - exposure delayed but not avoided | **No**  - selects sicker patients who place greater demands on health services |
| ***What are the human rights benefits?*** | - promotes access to care and treatment for those at greatest risk of illness | - Limits harm of exposure to toxic drugs | - all individuals <200 are equally eligible  - supports progressive realization (all patients will eventually meet criteria)  - unclear whether rationing in this way leads to community (health system) benefits |
| ***What are the human rights burdens?*** | - allows the development of stigmatizing illnesses  - poor availability of CD4 in rural areas may lead to discrimination by delaying ART initiation for the poorest | - unfavourable harm/benefit trade-off between limiting toxicity and treatment  - does not address causes of toxicity: all people will eventually be exposed | - inconsistent with the duty to immediately provide access to essential medicines  - places some patients at risk of disease (by allowing immune deterioration)  - blanket policy that rations care even in places that could enrol more patients  - may exhaust resources, and therefore limit access to health care, by focusing on the sickest |
| ***Are there less restrictive means to achieve the same objective?*** | **Yes**  - Raise threshold to <350 | **Yes**  - provide less-toxic drugs (eg TDF) | **Yes**  - decrease need to ration by increasing available resources  - reduce HIV programme spending by reducing costs elsewhere (drugs, lab investigations, human resources)  - increase capacity by adapting the model of delivery (out-of-clinic care or stable patients, nurse initiation of therapy)  - limit long-term need by scaling up prevention  - seek additional (international) resources to fund ART expansion |
| ***Are fair administrative procedures in place?*** | **No**  - inadequate assessment of latest evidence | **No**  - inadequate assessment of latest evidence | **No**  - rationale implied rather than stated  - public consultation lacking |
| ***Overall, is the policy the optimal approach to the problem?*** | **No**  Policy restricts treatment for those who could benefit, misses opportunities to minimize defaulting, and may promote stigma | **No**  Using less toxic drugs (TDF) would limit toxicity without limiting access to treatment. This would require a policy to procure less-expensive (generic) sources of TDF. | **No**  Reasons for rationing should be addressed |
