# Supplementary material for: When to start antiretroviral therapy in resource-limited settings: a human rights analysis
Source: BMC Int Health Hum Rights. 2010 Mar 31;10:6. doi: 10.1186/1472-698X-10-6 (PMC2864209; doi:10.1186/1472-698X-10-6)
Supplement: Additional file 2 — Recommendations for policy. This table provides a summary of recommendations for policy, taking into consideration the main issues identified by the human rights analysis. [file 1472-698X-10-6-S2.DOC]

**Table S2: Recommendations for policy**

________________________________________________________

Promote transparency in decision-making

- *Public discourse involving relevant stakeholders*

Move towards increased threshold for initiation (350 cells/μl)

- *Phased implementation to build support and learn lessons*
- *Clear, short-term targets for nationwide policy adoption*

Implement supportive policies to improve efficiency in expanded access to care

- *Task-shifting*
- *Decentralized care to clinic level*

Pilot new models of delivery

- *fast-track procedures and out-of-facility care for stable patients*

Reduce drug prices

- *Use policy measures such as compulsory licensing to secure more affordable sources of antiretrovirals*

Make available less toxic drugs

- *Include less-toxic regimens in EDL and national guidelines*

Promote human rights considerations in public health decision-making

________________________________________________________
